# Purification, Structural Characterization, and Antibacterial Evaluation of Poly-γ-Glutamic Acid from *Bacillus subtilis*

**DOI:** 10.3390/polym18020172

**Published:** 2026-01-08

**Authors:** Gobinath Chandrakasan, Genaro Martin Soto-Zarazúa, Manuel Toledano-Ayala, Priscila Sarai Flores-Aguilar, Said Arturo Rodríguez-Romero

**Affiliations:** 1 Facultad de Ingeniería Campus Amazcala, Universidad Autónoma de Querétaro, Carr. Chichimequillas S/N, Km 1, Amazcala, El Marqués 76265, Mexico; priscila.flores@uaq.mx (P.S.F.-A.);; 2 Facultad de Ingenieria, Universidad Autonoma de Queretaro, Cerro de las Campanas, Queretaro 76010, Mexico

**Keywords:** poly-γ-glutamic acid, antimicrobial properties, FT-IR, HPLC, *Bacillus subtilis*

## Abstract

Extracellular poly-γ-glutamic acid (γ-PGA) produced by *Bacillus* species demonstrates significant antibacterial properties, positioning it as a promising candidate for diverse biomedical and industrial applications. This study focused on molecular identification of *Bacillus subtilis* using Polymerase Chain Reaction (PCR) and evaluated the initial production of γ-PGA from a novel biological source of *Bacillus subtilis*. Shake flask fermentation was utilized for γ-PGA production, with three distinct growth media (Tryptic, MRS, and Mineral medium) assessed for their efficiency in polymer yield. Characterization of γ-PGA was conducted through FT-IR, HPLC, and GC-MS analyses. FT-IR spectroscopy confirmed the presence of characteristic functional groups such as carbonyl, amide, and hydroxyl groups. HPLC and GC-MS analyses provided insights into the polymer’s purity and molecular composition, highlighting components like methyl esters, hexanoic acid, and monomethyl esters. Furthermore, the study quantified γ-PGA production during a four-day shake flask fermentation period. These findings contribute significantly to bacterial characterization, optimization of fermentation processes, and the exploration of γ-PGA’s potential as an antibacterial agent. Future research directions include refining purification techniques to enhance γ-PGA’s antibacterial efficacy and expanding its applications across various fields.

## 1. Introduction

Poly-γ-glutamic acid (γ-PGA) is a biopolymer of increasing interest due to its remarkable properties, including biodegradability, water solubility, non-toxicity, and biocompatibility, which make it suitable for diverse applications in pharmaceuticals, cosmetics, agriculture, and food industries [1,2]. This extracellular biopolymer is predominantly synthesized by various *Bacillus* species and is known for its potential as a versatile biomaterial with applications ranging from drug delivery systems to functional food additives [3]. Principally noteworthy is its role as an effective antibacterial agent, exhibiting potential against both Gram-positive and Gram-negative pathogens [4]. The increasing demand for γ-PGA as a bioactive compound underscores the importance of developing efficient purification methods that preserve its antibacterial properties while ensuring high purity and yield. The extraction and purification of γ-PGA from microbial fermentation broth are crucial steps that determine its purity, functionality, and efficacy in applications. Several extraction methods have been explored, including precipitation, ultrafiltration, and chromatography, each influencing the yield and properties of the extracted γ-PGA [5]. Understanding and optimizing these extraction techniques are essential to harnessing the full potential of γ-PGA in different industrial sectors.

The versatile applications of γ-PGA stem from its unique molecular structure and physico-chemical properties. Characterization techniques such as FTIR, HPLC and GCMS play pivotal roles in elucidating the molecular structure, composition, and purity of γ-PGA [6]. These techniques provide insights into its chemical structure and functional groups, facilitating the development of tailored extraction and purification protocols. FTIR provides insights into the molecular structure and functional groups present in the polymer, elucidating its chemical composition and confirming its purity [7]. HPLC facilitates the quantitative analysis of γ-PGA and its related compounds, ensuring the accuracy of purification processes and determining the yield of the final product [8]. GC-MS complements these techniques by identifying the specific molecular components of γ-PGA, thereby confirming its identity and assessing any chemical modifications during purification [9]. This research contributes to the advancement of biotechnological applications of γ-PGA by offering insights into its purification using *Bacillus* species characterized by FTIR, HPLC, and GC-MS. The findings are expected to facilitate the development of novel antimicrobial agents with broad-spectrum activity and minimal ecological impact, catering to the growing demand for sustainable bioproducts in various industrial sectors.

Consequently, this investigation has demonstrated bacterial morphological and molecular characterization by *Bacillus* species. Then, γ-PGA produced by shake flask fermentation using a new biological source of *Bacillus suptilis* using optimizing three different media for efficient production of γ-PGA polymer. Extracted biopolymer characterized by FT-IR, HPLC and GCMS analysis. The final product of biopolymer will be evaluated antibacterial activity.

## 2. Materials and Methods

### 2.1. Bacterial Strain and Culture Conditions

*Bacillus subtilis* is a rod-shaped, Gram-positive bacterium commonly found in soil. The strain used in this study was isolated from soil as previously described [10]. *Bacillus subtilis* grow in the mesophilic temperature ranging from 25 to 35 °C. The bacterial strain used in this study was *Bacillus* species (Submitted in NCBI). The strain was maintained on nutrient agar plates and inoculated into 50 mL of liquid nutrient broth. The culture was incubated at 37 °C with shaking at 200 rpm for 24 h as the primary inoculum. This culture was then used to inoculate the production medium. Working stocks of bacterial cultures were maintained frozen at −80 °C in 20% glycerol suspensions, while for immediate use, cells were kept at 4 °C on Luria–Bertani agar plate containing (in g/L): peptone, 10; yeast extract, 5; NaCl, 10; agar, 23.

### 2.2. Production of Extracellular γ-PGA

Extracellular poly γ-PGA was produced using a modified method described by [11]. Bacterial isolates that produced high quantities of γ-PGA polymer were selected from isolation plates. These isolates were transferred to isolation broth medium and cultured in 250 mL Erlenmeyer flasks. The flasks were incubated at 37 °C for 2 days with shaking at 150 rpm. After the incubation period, bacterial growth was assessed by measuring the absorbance at 550 nm (A550). The effect of three different media such as Tryptic medium, MRS medium and Mineral medium (E-medium)-Selective medium for further study. (in g L^−1^): Glutamic acid-20; Citric acid-12; Glycerol-80; NH_4_Cl-7; MgSO_4_·7H_2_O-0.5; FeCl_3_·6H_2_O-0.04; K_2_HPO_4_-0.5; CaCl_2_·2H_2_O-0.15; MnSO_4_·H_2_O-0.04; ZnSO_4_·7H_2_O-0.35 has been studied. After incubation an incubation period, cells were removed by centrifugation, and the supernatant containing γ-PGA was collected and used for further precipitation. The extraction of γ-PGA was carried out using ammonium sulfate precipitation [12].

### 2.3. Molecular Size Estimation of Crude PGA

The molecular weight of crude γ-PGA was estimated using sodium dodecyl sulfate–polyacrylamide gel electrophoresis (SDS-PAGE), following the protocol described by [13]. Briefly, samples of c-PGA were loaded onto a polyacrylamide gel and subjected to electrophoresis under denaturing conditions. To visualize the acidic polymer, Alcian blue 8GX staining was employed, which selectively binds to polyanionic substances such as γ-PGA. This staining method provides clear visualization of γ-PGA bands within the gel matrix.

### 2.4. Characterization FTIR, HPLC and GC–MS Analysis

The γ-PGA used for all characterization analyses (FTIR, HPLC, and GC–MS) was obtained from the ammonium sulfate-precipitated fraction. Ethyl acetate extraction was used only for isolating low-molecular-weight secondary metabolites [14]. The ammonium sulfate-precipitated fraction was characterized by a Perkin Elmer spectrophotometer (PerkinElmer, Inc. Shelton, CT, USA) to determine the FTIR spectra of the attached functional groups in a scanning range of 0 to 4000 cm^−1^ with a resolution of 4 cm^−1^. Quantitative determination of γ-PGA was performed using HPLC. Prior to analysis, γ-PGA samples were hydrolyzed into L-glutamic acid monomers. A calibration curve was established using standard solutions of L-glutamic acid at known concentrations. The γ-PGA content in each sample was calculated based on the corresponding peak areas and the linear regression equation derived from the standard curve. Gas chromatography–mass spectrophotometry (GC-MS) was employed for the analysis of active constituents in *Bacillus* sp. Bacterial extracts using a GCMS-QP2010 Plus gas chromatograph (Shimadzu, Kyoto, Japan) were interfaced with a mass spectrometer [15]. The sample was introduced into a glass injector working in split mode with helium as the carrier gas and a linear velocity pressure of 81.7 kPa. The following conditions were used: Rtx-5 MS fused silica capillary column (30 m × 0.25 mm.i.d. × 0.25 µm film thickness). The following temperatures were used: column oven temp. 80.0 °C, injection temp. 270.00 °C. The constituents were identified using commercial libraries [16].

### 2.5. Antibacterial Assay

Extracts of *Bacillus* sp. active compounds were evaluated against both Gram-positive and Gram-negative bacteria. Bacterial strains *E. coli* (MTCC-2622), *S. aureus* (MTCC-96), *B. subtilus* (MTCC-2387), *P. mirabilis* (MTCC-1429), *E. faecalis* (MTCC-3159), and *P. stutzeri* (MTCC-4831) were obtained from microbial-type culture collection and Gene Bank (https://mtccindia.res.in, accessed on 24 September 2018). They were sub-cultured in nutrient broth for 24 h at 30 °C. For biological activity, each strain was swabbed consistently into individual nutrient agar plates using sterile cotton swabs [17]. With a sterile micropipette, each extracted bacterial compound (*Bacillus* sp. fraction 5 was taken in concentrations of 25 µL, 50 µL, 75 µL, and 100 µL), was loaded into each well. Ampicillin solvent served as the positive control (10 µg/mL). After 3 min, sterilized paper disks were pressed lightly on the surface of pathogenic plates. The doses were selected based on preliminary data obtained from earlier studies. After 24 h incubation at 37 °C, the different levels of zone of inhibition (ZOI) were measured.

### 2.6. Statistical Analysis

All experiments were performed in triplicate, and data are presented as mean ± standard deviation (SD). Statistical analysis was performed using [mention statistical tests] with *p* < 0.05 considered statistically significant

## 3. Results

### 3.1. Production of γ-PGA

**Figure 1:**
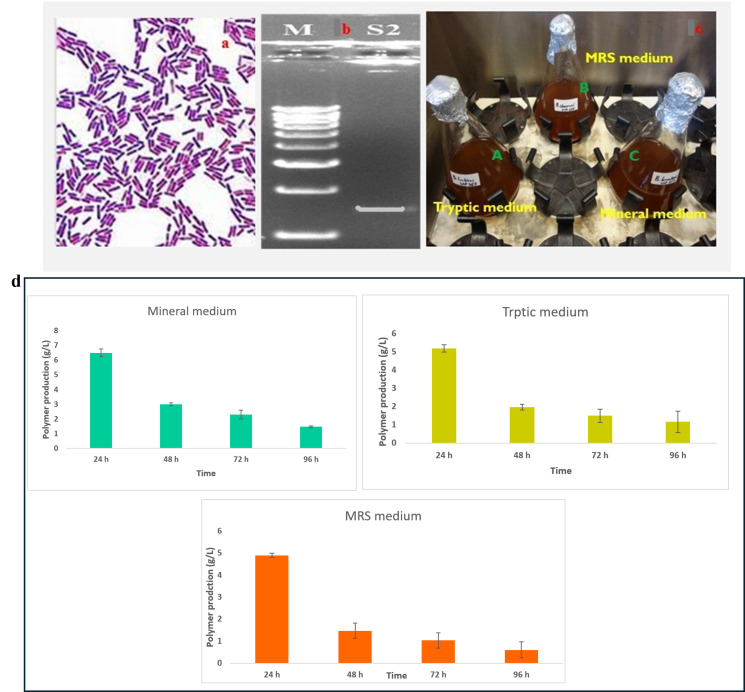
Characterization and selection of *Bacillus* sp. for γ-PGA production: (**a**)—Gram staining of the isolated *Bacillus* sp. observed under 100× magnification and (**b**)—molecular characterised by PCR (1450 bp) for further details see the Supplementary Materials S1; (**c**)—Optimization of three different medium for Gamma-PGA production (A-Tryptic medium, B-MRS medium and C-Mineral medium (E-medium)-Selective medium for further study). (**d**)—Polymer production (g/L) by the strain cultivated in Mineral, Tryptic, and MRS media at 24, 48, 72, and 96 h. Polymer synthesis peaked at 24 h in all media, with the highest yield observed in Mineral medium (≈6.8 g/L). Production steadily declined over time, with Mineral consistently supporting greater polymer accumulation compared to Tryptic and MRS. Values represent measured concentrations (g/L) at each time point.

The *Bacillus* species culture successfully produced extracellular γ-PGA under optimized conditions. After 72 h of fermentation, the culture supernatant was harvested, and γ-PGA was extracted and purified (Figure 1). The production of polymer (g/L) by the strain grown in three different media namely Mineral, Tryptic, and MRS—over four incubation periods (24, 48, 72, and 96 h) have been evaluated. Polymer production was highest at 24 h in all media and declined progressively with longer incubation times. At 24 h, the Mineral medium supported the greatest polymer yield (≈6.8 g/L), followed by Tryptic (≈5.4 g/L) and MRS (≈4.9 g/L). By 48 h, production decreased substantially across all media, with Mineral still maintaining the highest yield (≈2.9 g/L). At 72 h and 96 h, polymer concentrations continued to drop, reaching minimal levels in Tryptic and MRS by 96 h. Throughout the experiment, the Mineral medium consistently outperformed the other media, showing the most efficient support for polymer synthesis. These results indicate that polymer production is time-dependent, peaking early in the growth cycle, and that Mineral medium enhances polymer synthesis compared to Tryptic and MRS formulations (Figure 1d).

### 3.2. Structural Characterization of γ-PGA

**Figure 2:**
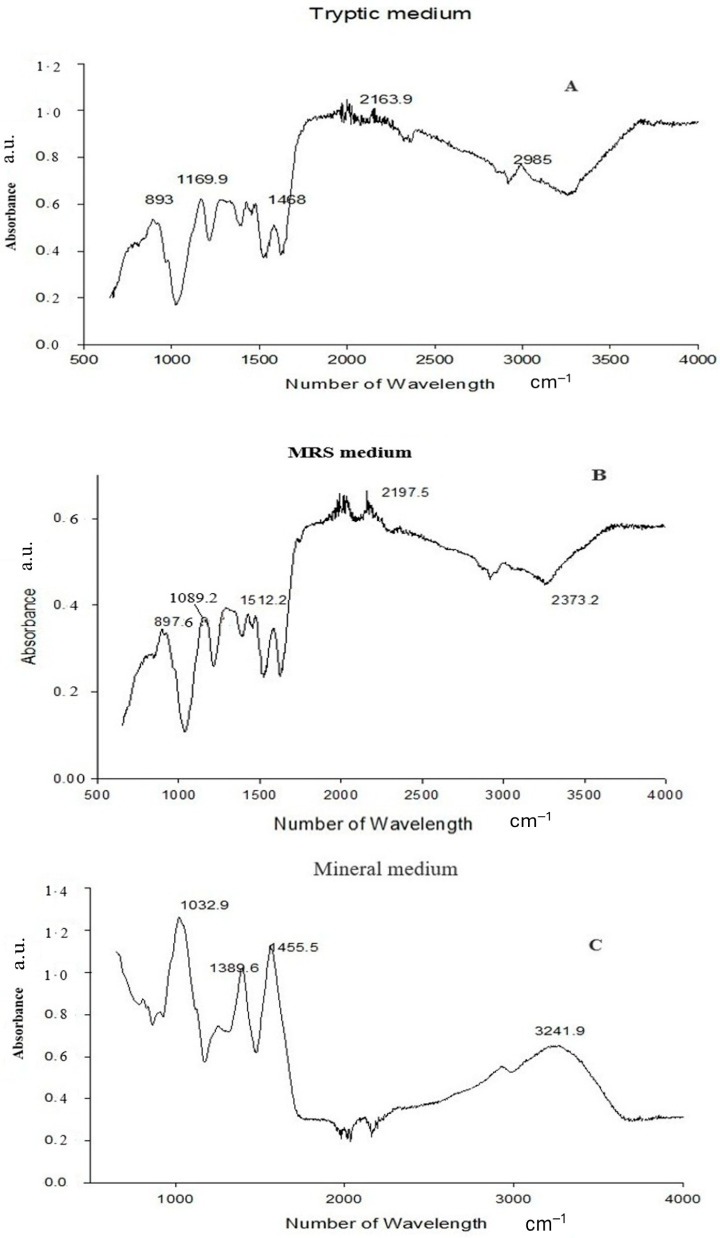
FTIR spectra of the biopolymer produced in (**A**) Tryptic medium, (**B**) MRS medium, and (**C**) Mineral medium. The analysis identifies the characteristic functional groups of PGA.

**Figure 3:**
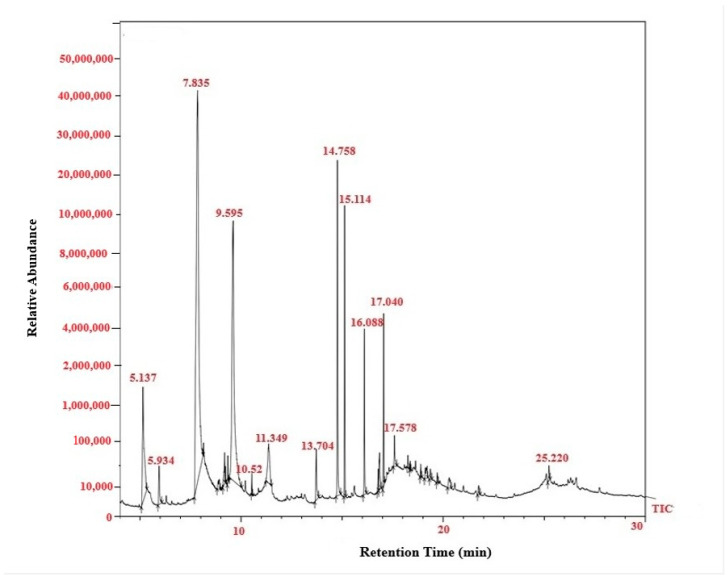
GC–MS total ion chromatogram (TIC) of γ-PGA produced by *Bacillus subtilis*. The chromatogram shows the retention profile of the derivatized γ-PGA fractions, with major peaks corresponding to the characteristic fragments of glutamic acid monomers and associated compounds detected within 10–30 min.

FTIR analysis clearly confirmed PGA synthesis in all three-culture media. The presence of the characteristic amide I (1600–1640 cm^−1^) and amide II (1390–1410 cm^−1^) bands—diagnostic for polyamide structures—verified the formation of the γ-glutamyl backbone typical of PGA [18]. A broad absorption between 3400 and 3460 cm^−1^, corresponding to O–H/N–H stretching vibrations, further supported the presence of hydroxyl and amide functional groups inherent to the polymer [19,20]. In addition to these core PGA features, medium-specific spectral variations were observed. PGA produced in Tryptic medium showed additional peaks at 893, 1169.9, 1468, 2163.9, and 2985 cm^−1^, while the MRS-derived polymer exhibited peaks at 897.6, 1099.2, 1512.2, 2197.5, and 2373.2 cm^−1^. The Mineral medium spectrum also included carbonyl-associated absorptions at 1132–1138 cm^−1^. These slight shifts and differences in peak intensities reflect medium-dependent structural variation, a common phenomenon in microbial PGA production, likely arising from changes in protonation state, side-chain interactions, or secondary-structure organization [21]. Overall, the FTIR profiles demonstrate that all three media successfully supported PGA synthesis, with compositional differences influencing fine structural characteristics of the final biopolymer. (Figure 2). Quantitative HPLC analysis confirmed the presence and concentration of γ-PGA in the purified sample. The analysis yielded a final γ-PGA concentration of 6.8 g/L, indicating efficient polymer production under the tested conditions, similarly correlated with Altun work [22]. The chromatographic profile showed a distinct and well-defined peak eluting between 10 and 32 min, which aligns with the retention time range established from the γ-PGA standard calibration curve [23] (Table 1). This correspondence between the sample peak and the standard validates the identity of the polymer detected in the chromatogram. The broad but characteristic elution pattern is consistent with γ-PGA’s polydisperse nature, where varying molecular weights can result in a wider retention time distribution. Together, these results confirm not only the presence of γ-PGA but also provide a reliable quantitative estimate of its production yield in the purified extract [24,25]. GC–MS Based Confirmation of γ-PGA Composition, because intact γ-PGA is a high-molecular-weight, highly polar biopolymer, it cannot be directly analyzed by GC–MS, which is optimized for volatile, low-molecular-weight compounds. Therefore, the GC–MS results reflect the composition of the monomers released after hydrolysis and derivatization rather than the polymer itself. Prior to GC–MS analysis, γ-PGA was subjected to acid hydrolysis, which cleaved the γ-amide bonds and released its constituent L- and D-glutamic acid monomers. These monomers were subsequently derivatized to increase volatility and thermal stability, enabling their detection by GC–MS (Data not showed) [26]. The chromatograms showed clear peaks corresponding to derivatized glutamic acid, confirming that the purified product consists of glutamate units—the defining structural feature of γ-PGA [27]. Detection of both D- and L-glutamic acid derivatives is consistent with the expected stereochemical composition of microbial γ-PGA. Thus, although GC–MS does not detect intact γ-PGA, the identification of its characteristic monomeric products provides indirect but strong evidence supporting the polymer’s chemical identity and purity (Figure 3).

### 3.3. SDS–PAGE of the c-PGA

**Figure 4:**
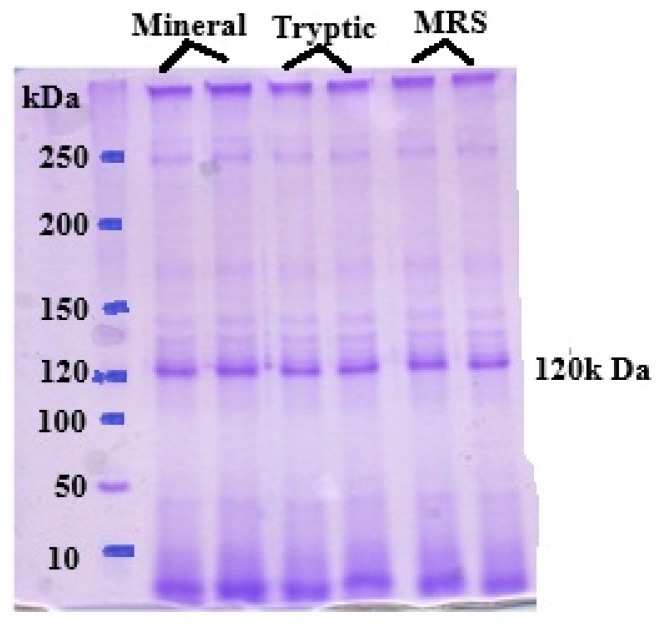
SDS-PAGE analysis of γ-PGA produced by *Bacillus subtilis* in an optimized medium reveals that the PGA typically appears as high molecular 120 KDa.

The purification process involved ammonium sulfate precipitation followed by dialysis. SDS-PAGE analysis of the purified γ-PGA revealed a single prominent band corresponding to γ-PGA, confirming its purity (Figure 4). *B. subtilis* strain C1 synthesized high-molecular-weight crude γ-PGA, with an estimated size exceeding 650 kDa, across all tested media formulations. The highest molecular mass was observed when the strain was cultivated in the optimized medium, suggesting favorable conditions for the production of large polymer chains. The molecular weight of c-PGA is particularly important for its functional applications, including antimicrobial activity. Different applications may require specific molecular size ranges to optimize performance, such as enhancing viscosity, film-forming capacity, or interaction with microbial cell surfaces. The consistent production of high-molecular-weight c-PGA by strain C1 underscores its potential as a candidate for developing antimicrobial formulations and other biotechnological applications.

### 3.4. Antibacterial Effect

**Figure 5:**
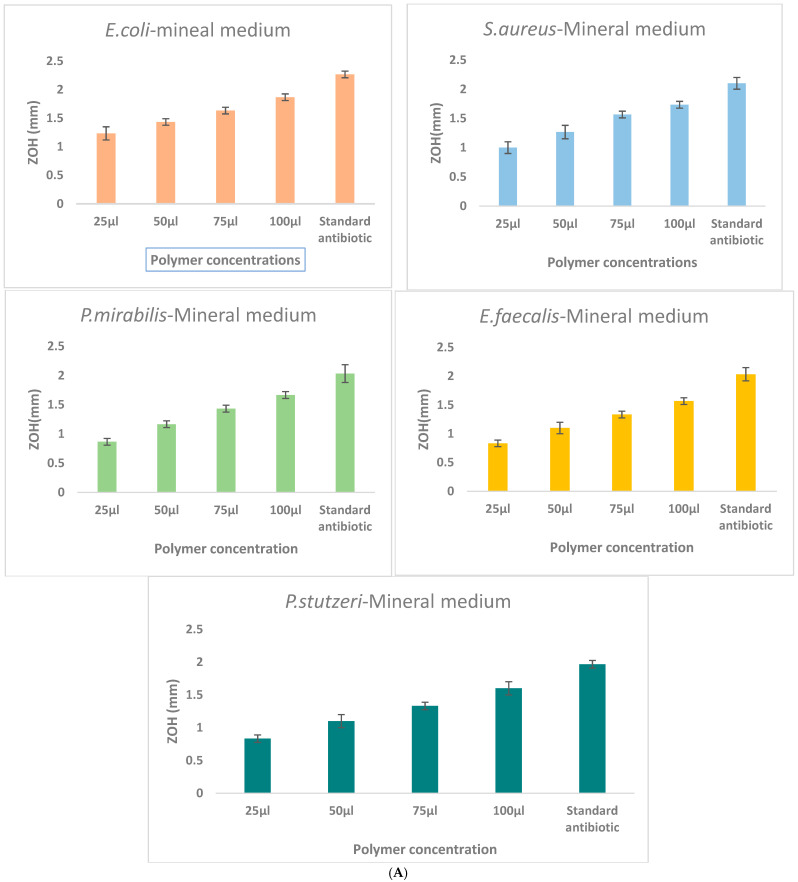

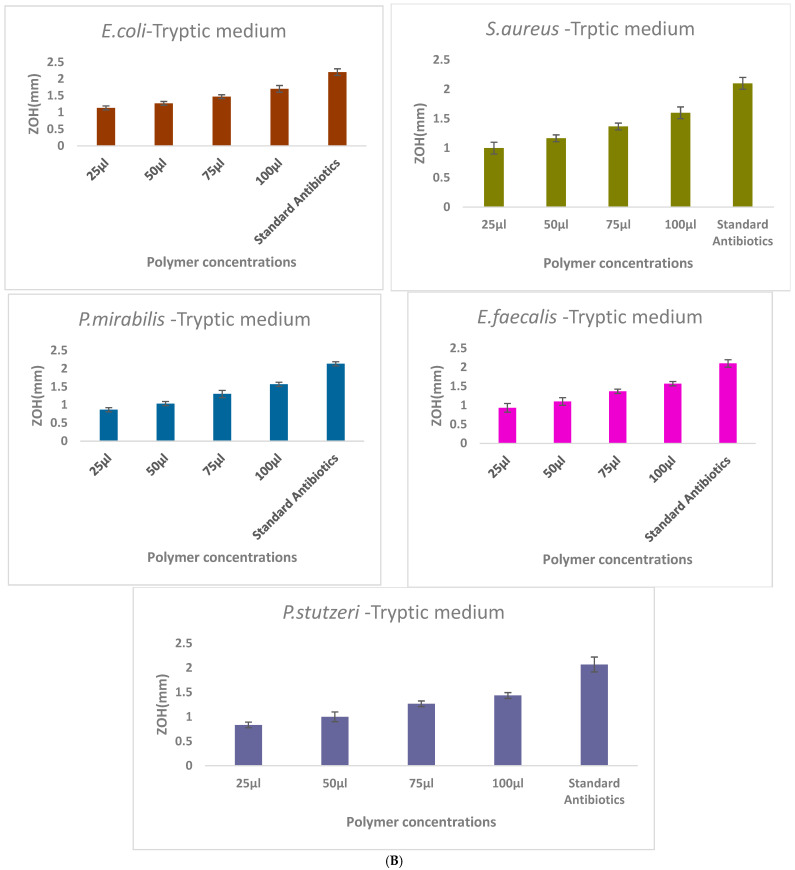

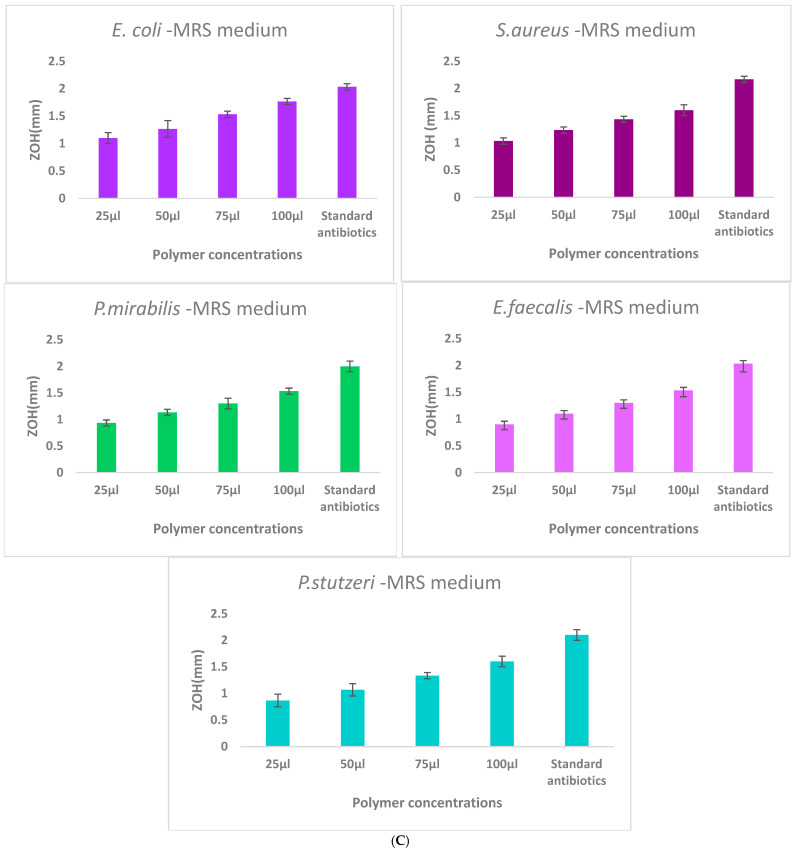
Comparison of γ-PGA production in three different culture media: (**A**)—Mineral medium (E-medium); (**B**)—Tryptic medium, and (**C**)—MRS medium. The selectively extracted (γ-PGA) polymer from the mineral medium (E-medium) exhibited enhanced antibacterial activity compared to polymers obtained from the other two media, suggesting that medium composition influences the biochemical properties and antibacterial mechanism of γ-PGA. Zone of inhibition (ZOH, mm) produced by γ-PGA concentrations of the extract (25, 50, 75, and 100 μL) compared with the ampicillin standard antibiotic control against *E. coli*, *S. aureus*, *P. mirabilis*, *E. faecalis*, and *P. stutzeri*. Each value represents mean inhibition zones from triplicate experiments.

The antibacterial activity of γ-PGA was assessed against *E. coli*, *S. aureus*, *B. subtilus*, *P. mirabilis*, *E. faecalis*, and *P. stutzeri* using the agar well diffusion method. A high level of inhibition was showed in the *Staphylococcus aureus* and *Escherichia coli*, the remaining three pathogens had moderate level of inhibition with clear zones of inhibition observed around wells containing γ-PGA solutions (Figure 5). The diameter of inhibition zones increased with increasing concentrations of γ-PGA, indicating dose-dependent antibacterial efficacy.

## 4. Discussion

Extracellular poly-γ-PGA, a naturally occurring biopolymer synthesized predominantly by *Bacillus* species, has attracted considerable interest due to its biocompatibility, biodegradability, and broad range of industrial applications [28]. In the present study, γ-PGA was successfully isolated and purified from the culture supernatant of a *Bacillus* strain, with a focus on optimizing both production and purification processes. Fermentation under controlled conditions yielded a high concentration of γ-PGA, demonstrating the strain’s strong biosynthetic capability [29,30].

Fourier-transform infrared spectroscopy (FTIR) provided valuable insights into the structural composition of γ-PGA. The FTIR spectrum exhibited characteristic absorption bands at 1640 cm^−1^ (amide I) and 1530 cm^−1^ (amide II), indicative of the peptide bonds in the polymer backbone [31]. The FTIR spectral analysis revealed characteristic absorption bands indicative of glutamic acid residues interconnected via amide bonds, thereby confirming the polymeric structure of γ-PGA. Specifically, the presence of strong amide I (C=O stretching) and amide II (N–H bending) bands supports the formation of peptide linkages, which are consistent with the repetitive glutamyl units forming the γ-PGA backbone [32]. Additionally, the FTIR absorption bands observed at 2920 cm^−1^ and 2850 cm^−1^ correspond to the asymmetric and symmetric stretching vibrations of –CH_2_ groups, indicating the presence of aliphatic moieties within the γ-PGA structure [33]. These features, in conjunction with the characteristic amide bands, reinforce the identification of γ-PGA and provide insight into its molecular composition. The FTIR analyses collectively demonstrate that all three media supported successful γ-PGA biosynthesis, while also revealing medium-dependent structural variations at the functional-groups and conformational levels [34]. FTIR spectra from all samples consistently showed the characteristic amide I (1600–1640 cm^−1^) and amide II (1390–1410 cm^−1^) absorption bands, confirming preservation of the γ-glutamyl backbone regardless of the medium used [35]. However, additional medium-specific bands-such as the bands at 893–2985 cm^−1^ in the Tryptic-derived polymer, 897–2373 cm^−1^ in the MRS-derived polymer, and 1132–1138 cm^−1^ band in the Mineral-derived polymer-indicated subtle structural differences. These variations likely reflect changes in protonation state, hydrogen-bonding patterns, and secondary-structure organization, all of which are known to be influenced by nutrient composition, ionic strength, and carbon/nitrogen availability in microbial γ-PGA biosynthesis [36]. Importantly, these shifts represent conformational or interaction-based modifications rather than alterations to the fundamental polymer backbone.

HPLC analysis was conducted to evaluate the composition and purity of γ-PGA produced by *Bacillus subtilis* in three different media: MRS, Mineral, and Tryptic soy broth. The HPLC chromatograms revealed distinct fractions corresponding to γ-PGA, with retention times consistent with γ-PGA standards, confirming the accuracy and reliability of the quantification method [37]. In cultures grown with MRS medium, seven prominent peaks were observed, indicating the presence of multiple γ-PGA fractions. These peaks suggest a relatively uniform but diverse molecular composition of the polymer under nutrient-rich conditions. In mineral medium, nine distinct fractions were detected, the highest number among the tested conditions. This suggests that the limited nutrient composition in the mineral medium may have promoted a broader distribution of γ-PGA molecular variants, possibly due to variable chain lengths or minor by-products associated with altered metabolic pathways. The tryptic soy medium also yielded seven γ-PGA peaks, similar to the MRS medium, though subtle differences in peak intensity and retention times suggest variations in the polymer structure or molecular weight distribution.

The concentration of γ-PGA measured in this study (6.8 g/L) underscores its efficient production and purification. GC-MS analysis of the hydrolyzed γ-PGA confirmed glutamic acid (Glu) as the predominant monomeric unit, consistent with the established chemical structure of γ-PGA. This finding aligns with previous studies reporting that γ-PGA is primarily composed of D- and L-glutamic acid residues linked via γ-amide bonds [38,39]. The identification of Glu not only verifies the polymer’s integrity but also affirms the effectiveness of the hydrolysis and detection protocols employed in this study.

Mineral medium provided the highest γ-PGA production and yielded polymers with superior antibacterial activity compared with Tryptic and MRS formulations. The maximum yield of ≈6.8 g/L at 24 h exceeds or matches many recently reported values, including those of optimized systems such as *B. subtilis* BL53 [40]. The γ-PGA produced in Mineral medium also exhibited stronger, dose-dependent inhibition against *S. aureus* and *E. coli*, demonstrating that medium composition affects both polymer quantity and functional performance. These results identify Mineral medium as the most efficient option for high-yield and high-functionality γ-PGA production, supporting its suitability for antimicrobial and biotechnological applications.

The broad elution range is characteristic of γ-PGA polydispersity and supports the presence of molecules spanning multiple molecular-weight fractions. Complementary GC–MS characterization, performed after hydrolysis and derivatization, identified both L- and D-glutamic acid as the primary monomeric components, confirming the expected stereochemical and compositional identity of microbial γ-PGA. Together, these results indicate that while medium composition influences fine molecular features of the polymer (e.g., functional-group environment, secondary structure), the core γ-PGA structure remains unchanged, demonstrating that culture conditions modulate polymer conformation rather than its backbone chemistry.

The purification method comprising ammonium sulfate precipitation followed by dialysis proved effective in removing proteins and other contaminants, as confirmed by SDS-PAGE analysis, which indicated a high degree of purity [41]. The yield and purity achieved in this study are comparable to or even greater than those reported in similar studies, highlighting the reliability and efficiency of the proposed approach [42]. High-purity γ-PGA is particularly important for applications where product quality is paramount, such as in pharmaceuticals, food formulations, and cosmetic products [43,44].

A significant aspect of this study was the investigation of the antibacterial potential of γ-PGA against clinically relevant bacterial pathogens, *S. aureus* and *E. coli*. The antibacterial activity was assessed using the agar well diffusion method, where γ-PGA exhibited clear and measurable zones of inhibition around the wells containing γ-PGA solutions. This result indicates that γ-PGA possesses intrinsic antibacterial properties capable of suppressing both Gram-positive and Gram-negative bacteria, consistent with previous reports demonstrating its bioactivity [45,46]. The mechanism behind this antimicrobial effect is not fully understood but is hypothesized to involve the interaction of the negatively charged γ-PGA with positively charged sites on bacterial cell surfaces, potentially disrupting membrane integrity or interfering with nutrient uptake. Moreover, γ-PGA’s highwater solubility, biodegradability, and non-toxic nature make it an attractive candidate for biomedical applications such as wound dressings, antioxidants, cytotoxic properties drug delivery matrices, and antimicrobial coatings [47,48].

**Figure 6:**
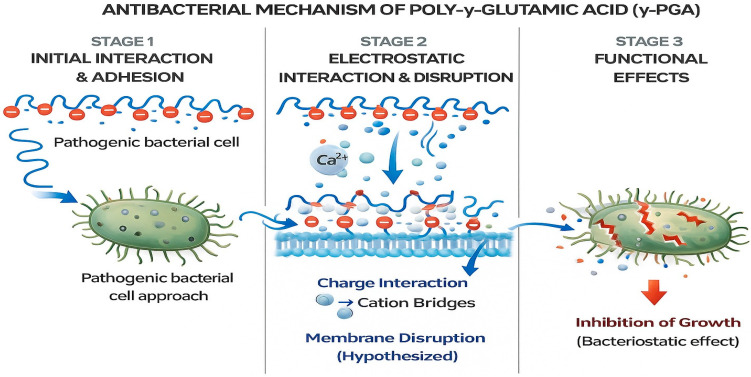
Schematic illustrates the proposed three-stage antibacterial action of γ-PGA. Stage 1: Initial interaction and adhesion-the anionic γ-PGA polymer interacts with the approaching pathogenic bacterial cell surface. Stage 2: Electrostatic interaction and membrane disruption-γ-PGA forms electrostatic interactions, including cation-bridging (e.g., Ca^2+^), leading to destabilization of the bacterial membrane. Stage 3: Functional effects-membrane perturbation and physicochemical stress result in inhibition of bacterial growth, suggesting a primarily bacteriostatic effect.

The mechanism underlying the antibacterial activity of γ-PGA is likely multifactorial and may involve several biological interactions. One proposed mechanism is the disruption of bacterial cell membranes through electrostatic interactions between the anionic γ-PGA and cationic components of the bacterial envelope (Figure 6), leading to increased membrane permeability and eventual cell lysis [49]. Another important consideration is the role of γ-PGA in modulating microbial biofilms. Previous studies have shown that γ-PGA can prevent biofilm formation or destabilize pre-formed biofilms, particularly in *Staphylococcus aureus* and *Escherichia coli*, by altering surface adhesion properties or penetrating the extracellular matrix [50]. This anti-biofilm activity is especially valuable in clinical settings, where biofilm-associated infections are resistant to conventional antibiotics [51]. Further studies are warranted to elucidate the specific mechanisms responsible for the observed antibacterial effects and to optimize γ-PGA formulations for enhanced antimicrobial potency and rheological properties [52]. Comparative analysis of γ-PGA with other biopolymers and synthetic antimicrobial agents highlights its unique properties, including biocompatibility and environmentally friendly production processes. Unlike many synthetic antibiotics that may pose risks of cytotoxicity, environmental persistence, and development of resistance, γ-PGA offers a biocompatible and environmentally friendly alternative [53]. As a naturally occurring, biodegradable polymer, γ-PGA exhibits low toxicity to human cells and is readily broken down by microbial enzymes into non-toxic glutamic acid monomers, making it safe for biomedical and environmental applications [54].

These characteristics not only minimize ecological impact but also reduce the likelihood of adverse side effects, which are common with many conventional antimicrobials. The sustainability of γ-PGA-based antimicrobial systems is particularly appealing in the context of rising antibiotic resistance and environmental contamination by pharmaceutical residues. Its production from renewable microbial sources and its complete biodegradability make γ-PGA a promising material for use in wound dressings, food preservation, drug delivery, and agricultural biocontrol strategies [55,56]. The findings of this study contribute to the growing body of research on γ-PGA and underscore its potential as a versatile biopolymer with diverse industrial applications. Future research directions could focus on optimizing production processes to enhance yield and purity, exploring additional biological activities such as antiviral or wound-healing properties, and investigating synergistic effects with conventional antibiotics.

## 5. Conclusions

This study successfully identified a novel strain of *Bacillus subtilis* and demonstrated its capacity to produce γ-PGA under shake flask fermentation using various growth media. Among the tested media, differential yields highlighted the influence of nutrient composition on γ-PGA biosynthesis. Comprehensive structural characterization through FT-IR, HPLC, and GC-MS confirmed the polymer’s identity, purity, and the presence of functional groups and compounds relevant to its biological activity. Notably, the antibacterial assessment suggests that γ-PGA holds significant potential as a natural antimicrobial agent. These findings advance our understanding of γ-PGA production and structure–function relationships, supporting its further development for biomedical and industrial applications. Future studies should focus on scaling up production, enhancing purification strategies, and conducting in-depth evaluations of antibacterial mechanisms and spectra.

## Figures and Tables

**Table 1:** Polyglutamic acid biopolymer HPLC fractions obtained from bacterial culture grown in MRS medium (Run 1), Mineral medium (Run 2) and Tryptic medium (Run 3). Retention time, peak width, area, height, and percentage area of the detected compounds are presented, indicating the relative abundance of each eluted fraction.

Run	Peak	Retention Time (min)	Peak Width (min)	Area (mAU·s)	Height (mAU)	Area (%)	Normalized Area (cm^2^)
**Run 1**	1	10.772	0.3548	263.52	15.84	1.249	1.25
	2	11.784	0.7325	135.55	4.58	0.582	0.58
	3	18.725	0.5322	734.66	114.49	2.449	2.45
	4	19.772	1.2871	14,214.44	150.42	72.426	72.43
	5	22.331	0.5531	210.62	7.77	0.300	0.30
	6	27.622	0.7825	1352.33	23.56	7.473	7.47
	7	32.132	1.2492	4372.57	36.59	22.774	22.77
**Run 2**	1	9.133	0.5419	2401.91	36.72	1.038	1.04
	2	10.771	0.2103	149.95	18.10	0.099	0.10
	3	12.597	0.8358	152.67	16.20	0.072	0.07
	4	13.149	0.5678	6645.23	133.45	4.225	4.22
	5	16.478	0.7686	74,782.20	1764.55	12.264	12.26
	6	19.653	1.5388	45,743.80	1840.21	55.773	55.77
	7	24.442	1.2516	5775.29	201.29	8.468	8.47
	8	30.308	0.7701	1967.89	187.45	5.740	5.74
	9	32.550	2.6330	89,881.00	286.71	14.654	14.65
**Run 3**	1	10.261	0.6558	231.33	16.55	0.433	0.43
	2	12.321	0.9988	2391.78	54.38	3.165	3.17
	3	16.553	0.4675	8512.43	589.55	9.450	9.45
	4	19.656	1.0759	117,999.60	621.61	63.867	63.87
	5	22.116	1.8866	174.89	777.60	3.343	3.34
	6	26.550	0.9933	865,560.00	241.78	24.663	24.66
	7	30.944	1.7662	3665.90	183.99	14.664	14.66

## Data Availability

The original data used in this research are included in the article.

## Supplementary Materials

The following supporting information can be downloaded at: https://www.mdpi.com/article/10.3390/polym18020172/s1, Supplementary Material S1: PCR-Based Identification of *Bacillus subtilis* Using 16S rRNA Gene Sequencing.
