# Supplementary material for: Purification, Structural Characterization, and Antibacterial Evaluation of Poly-γ-Glutamic Acid from Bacillus subtilis
Source: Polymers (Basel). 2026 Jan 8;18(2):172. doi: 10.3390/polym18020172 (PMC12846282; doi:10.3390/polym18020172)
Supplement: Supplementary file 1 [file polymers-18-00172-s001.zip › polymers-4012697-supplementary.pdf]

Supplementary Material S1. PCR-Based Identification of *Bacillus subtilis* Using 16S rRNA Gene Sequencing

S1.1. DNA Extraction

Genomic DNA was extracted from freshly grown bacterial cultures using a standard phenol–chloroform extraction procedure. DNA integrity and concentration were verified by 1% agarose gel electrophoresis and spectrophotometric quantification.

S1.2. PCR Amplification of the 16S rRNA Gene

Molecular identification of the bacterial isolate was performed by amplifying the 16S rRNA gene using universal bacterial primers targeting conserved regions.

Primers Used

- **Forward primer (16S-20):** 5′-AGA GTT TGA TCC TGG CTC-3′
- **Reverse primer (16S-1390):** 5′-GAC GGG CGG TGT GTA CAA-3′

PCR Reaction Mixture (50 µL total volume)

Component	Volume
10× PCR buffer	5 µL
$MgCl_2$	5 µL
dNTP mix	1 µL
Primer 16S-20	0.4 µL
Primer 16S-1390	0.4 µL
Taq DNA polymerase	0.4 µL
Template DNA	5 µL
Nuclease-free water	32.8 µL

S1.3. PCR Cycling Conditions

PCR amplification was performed under the following thermal conditions:

1. **Initial denaturation:** 94 °C for 5 min
2. **Denaturation:** 94 °C for 30 s
3. **Annealing:** 50 °C for 30 s
4. **Extension:** 72 °C for 1 min
5. **Final extension:** 72 °C for 15 min

Total cycles: 35

PCR products were analyzed on a 1% agarose gel. A clear single band of approximately **1,450 bp** was obtained, corresponding to the expected size of the bacterial 16S rRNA gene.

S1.4. Sequencing and Identification

The purified PCR amplicon was subjected to Sanger sequencing. Resulting sequences were aligned using BLASTn against the NCBI nucleotide database.

- Sequence identity: **98–99% similarity to *Bacillus subtilis*** reference strains
- Phylogenetic placement: The isolate clustered within the *B. subtilis* clade in a distance-based phylogenetic tree, confirming species-level identity.

Run	Peak	Retention time (min)	Peak width (min)	Area (mAU·s)	Height (mAU)	Area (%)	Normalized area (cm²)
**Run 1**	1	10.772	0.3548	263.52	15.84	1.249	1.25
	2	11.784	0.7325	135.55	4.58	0.582	0.58

	3	18.725	0.5322	734.66	114.49	2.449	2.45
	4	19.772	1.2871	14,214.44	150.42	72.426	72.43
	5	22.331	0.5531	210.62	7.77	0.300	0.30
	6	27.622	0.7825	1,352.33	23.56	7.473	7.47
	7	32.132	1.2492	4,372.57	36.59	22.774	22.77
**Run 2**	1	9.133	0.5419	2,401.91	36.72	1.038	1.04
	2	10.771	0.2103	149.95	18.10	0.099	0.10
	3	12.597	0.8358	152.67	16.20	0.072	0.07
	4	13.149	0.5678	6,645.23	133.45	4.225	4.22
	5	16.478	0.7686	74,782.20	1,764.55	12.264	12.26
	6	19.653	1.5388	45,743.80	1,840.21	55.773	55.77
	7	24.442	1.2516	5,775.29	201.29	8.468	8.47
	8	30.308	0.7701	1,967.89	187.45	5.740	5.74
	9	32.550	2.6330	89,881.00	286.71	14.654	14.65
**Run 3**	1	10.261	0.6558	231.33	16.55	0.433	0.43
	2	12.321	0.9988	2,391.78	54.38	3.165	3.17
	3	16.553	0.4675	8,512.43	589.55	9.450	9.45
	4	19.656	1.0759	117,999.60	621.61	63.867	63.87
	5	22.116	1.8866	174.89	777.60	3.343	3.34
	6	26.550	0.9933	865,560.00	241.78	24.663	24.66
	7	30.944	1.7662	3,665.90	183.99	14.664	14.66

Comment 1: 1) The absorbance values on the y-axis of the FT-IR spectra (Fig. 2) are extremely low, with all readings falling below 0.1. The authors are requested to verify this dataset and confirm its accuracy.

Answer: now I revised the FTIR spectra for the accuracy

2) Additionally, it would be helpful to clarify whether the sample employed for FT-IR analysis was a dilute γ-PGA solution or an ultra-thin solid film.

Answer: A small amount of the biopolymer powder is placed directly onto the surface of an FTTR crystal (commonly made of zinc selenide). Pressure is then applied using a pressure clamp to ensure good, consistent contact between the sample and the crystal surface. The spectrum is acquired directly.

3) The authors should also provide a detailed explanation for the unusually low absorbance values observed in Fig. 2.

Answer: now I revised the FTIR spectra for the accuracy

Comment 2: In my opinion the Tables 1-3 are not correct in respect with units used. If the authors kept for all samples, the same conditions of measurements, they could made a comparative presentation with area measured in known units of cm2.

Answer: Thanks for comments, I modified table 1 the according to your suggestion.
